# New Potentially Active Pyrazinamide Derivatives Synthesized Under Microwave Conditions

**DOI:** 10.3390/molecules19079318

**Published:** 2014-07-03

**Authors:** Ondrej Jandourek, Martin Dolezal, Jiri Kunes, Vladimir Kubicek, Pavla Paterova, Matus Pesko, Vladimir Buchta, Katarina Kralova, Jan Zitko

**Affiliations:** 1 Department of Medicinal Chemistry and Drug Analysis, Faculty of Pharmacy, Charles University in Prague, Heyrovskeho 1203, Hradec Kralove 50005, Czech Republic; E-Mail: dolezalm@faf.cuni.cz; 2 Department of Inorganic and Organic Chemistry, Faculty of Pharmacy, Charles University in Prague, Heyrovskeho 1203, Hradec Kralove 50005, Czech Republic; E-Mail: kunes@faf.cuni.cz; 3 Department of Biophysics and Physical Chemistry, Faculty of Pharmacy, Charles University in Prague, Heyrovskeho 1203, Hradec Kralove 50005, Czech Republic; E-Mail: kubicek@faf.cuni.cz; 4 Department of Biological and Medical Sciences, Faculty of Pharmacy, Charles University in Prague, Heyrovskeho 1203, Hradec Kralove 50005, Czech Republic; E-Mails: pavla.paterova@fnhk.cz (P.P.); vladimir.buchta@fnhk.cz (V.B.); 5 Department of Environmental Ecology, Faculty of Natural Sciences, Comenius University, Mlynska Dolina CH-2, Bratislava 842 15, Slovakia; E-Mail: matus.pesko@gmail.com; 6 Institute of Chemistry, Faculty of Natural Sciences, Comenius University, Mlynska Dolina CH-2, Bratislava 842 15, Slovakia; E-Mail: kata.kralova@gmail.com

**Keywords:** aminodehalogenation reaction, antifungal activity, inhibition of photosynthetic electron transport, pyrazinamide, microwave-assisted synthesis, structure-activity relationships

## Abstract

A series of 18 *N*-alkyl substituted 3-aminopyrazine-2-carboxamides was prepared in this work according to previously experimentally set and proven conditions using microwave assisted synthesis methodology. This approach for the aminodehalogenation reaction was chosen due to higher yields and shorter reaction times compared to organic reactions with conventional heating. Antimycobacterial, antibacterial, antifungal and photosynthetic electron transport (PET) inhibiting *in vitro* activities of these compounds were investigated. Experiments for the determination of lipophilicity were also performed. Only a small number of substances with alicyclic side chain showed activity against fungi which was the same or higher than standards and the biological efficacy of the compounds increased with rising lipophilicity. Nine pyrazinamide derivatives also inhibited PET in spinach chloroplasts and the IC_50_ values of these compounds varied in the range from 14.3 to 1590.0 μmol/L. The inhibitory activity was connected not only with the lipophilicity, but also with the presence of secondary amine fragment bounded to the pyrazine ring. Structure-activity relationships are discussed as well.

## 1. Introduction

Pyrazinamide (PZA) is considered to be one of the most important drugs in the World. It has numerous uses and the pyrazine ring is also contained in many natural compounds. Nevertheless, its main field of use is still the treatment of tuberculosis (TB). The reason is clear—*Mycobacterium tuberculosis* is the most dreadful microorganisms in the World. Although the incidence of new TB cases has been falling slowly throughout the last decade, the danger is posed by the group of resistant strains [1]. There are currently two types of resistance. The first group consists of multidrug-resistant mycobacteria, which are resistant to all first-line antituberculotic agents (rifampicin/rifabutin, isoniazid, pyrazinamide, ethambutol, streptomycin). The second group is more treacherous because of its resistance to isoniazid and rifampicin combined with resistance to any fluoroquinolone used in the current therapy and to at least one of the three parenteral second-line antituberculotic drugs (amikacin, kanamycin, capreomycin) [2]. There was another category in the past called totally drug-resistant TB that was first observed in India. These strains were resistant to all known first- and second-line antituberculosis agents and posed a threat to epidemiologists all over the World. But they have practically disappeared with the discovery and approval of the new anti-tuberculosis agent bedaquiline for their therapy because it is active against the resistant *Mycobacterium tuberculosis* [3]. PZA is classified as the first-line antituberculotic agent that is unique for its therapeutic properties. It acts in acidic environment as a bactericidal sterilising drug that is able to kill the dormant forms of tubercle bacilli, especially in combination with rifampicin. These so called persistors are just situated *in nidus* where an acidic pH is typical [4].

On the contrary, the mechanism of action of PZA is still under the investigation by researchers all over the World. One of the first theories suggested was that it is necessary to activate PZA via the enzyme pyrazinamidase (nicotinamidase) (EC 3.5.1.19) to the active form pyrazinecarboxylic acid (POA). This acid molecule gets into the mycobacterial cell and causes the inner cell compartment acidification during the ionic efflux. These consequences lead to cellular death [5,6,7]. The gene encoding pyrazinamidase is known as *pncA* gene and its mutation leads to the resistance of mycobacteria to pyrazinamide [8].

Another theory propounded the inhibition of fatty acid synthase I (FAS I) (EC 2.3.1.85). This is the enzyme essential for synthesis of mycolic acids, which are vital for the mycobacterial cell wall integrity. Despite the fact that PZA is effective in this aspect, this mechanism was rejected by Boshoff who has proven that PZA caused only low inhibition [9]. PZA analogues that act as FAS I inhibitors are from the group of 5-chloropyrazinamide, esters of 5-chloropyrazinoic acid or POA and their derivatives [10,11,12].

A recent theory introduced by Zhang *et al.* proposed the inhibition of *trans*-translation as the main way of PZA action. This cellular process is essential for survival and virulence of mycobacteria and its interruption leads to fatal blockade of proteosynthesis. All these hypotheses were supported by *in vitro* experiments [13].

PZA and its derivatives have also many other useful applications, as they show antiviral, antifungal, antibacterial and as well as antineoplastic activities [14,15,16,17,18,19,20]. Several pyrazine derivatives were also found to inhibit photosynthetic electron transport (PET) in plant chloroplasts [21,22,23]. Using EPR spectroscopy it was found that the site of action of pyrazine-derived PET inhibitors is the intermediate D**^•^**, * i.e.*, tyrosine radical (Tyr_D_**^•^**) situated in the 161st position in D_2_ protein on the donor side of photosystem (PS) 2 [24,25]. Similar site of action in the photosynthetic apparatus of spinach chloroplasts was confirmed for 2-alkylsulfanylpyridine-4-carbothioamides [26] and substituted benzanilides and thiobenzanilides [27]. Further experiments with artificial electron donor 1,5-diphenylcarbazide (DPC) confirmed that PET between PS2 and PS1 was interrupted by 2,6-disubstituted pyridine-4-thiocarboxamides exclusively on the donor side of PS2 in the section between the primary electron donor of PS2 (H_2_O) and Z**^•^**/D**^•^** intermediate and the photosynthetic transport chain from P 680 to plastoquinone Q_B_ occurring on the acceptor side of PS2 was not damaged [25], while PET on the acceptor side of PS2 was partially damaged by 5-*tert*-butyl-6-chloro-*N*-(3-fluorophenyl)pyrazine-2-carboxamide and 5-*tert*-butyl-*N*-(3-hydroxy-4-chlorophenyl)pyrazine-2-carboxamide [25] or by *N*-benzylpyrazine-2-carboxamides [22]. Many structural variations of pyrazine compounds with herbicidal properties can be found in the patent literature focused on compounds with herbicidal activity that are useful for the control of unwanted vegetation [28,29,30].

A further aspect of this work is the use of microwave-assisted reactions. It is known that microwave-assisted syntheses have become into general awareness during the last decades and their applications are still in the focus of many researchers. These reactions usually provide higher yields, shorter reaction times and better chemical conversions in comparison with conventional heating methods. Moreover, sometimes the desired products can be obtained only by using the microwaves accelerated reactions [31]. If emerging side-products are detected, they can be eliminated by many separation methods such as chromatography or crystallization. The advantages of these reactions are explained by the interaction between microwaves and molecules themselves, neither vessel sides nor any solvents used. There is also the possibility of reaching temperatures higher than the boiling point of solvent, especially in over-pressurized systems. This has opened a new way for drug discovery and development [32]. We focus herein on the synthesis of pyrazine derivatives with aliphatic or alicyclic side chains and their antimycobacterial, antibacterial, antifungal and PET-inhibiting activities.

## 2. Results and Discussion

### 2.1. Chemistry

**Scheme 1:**
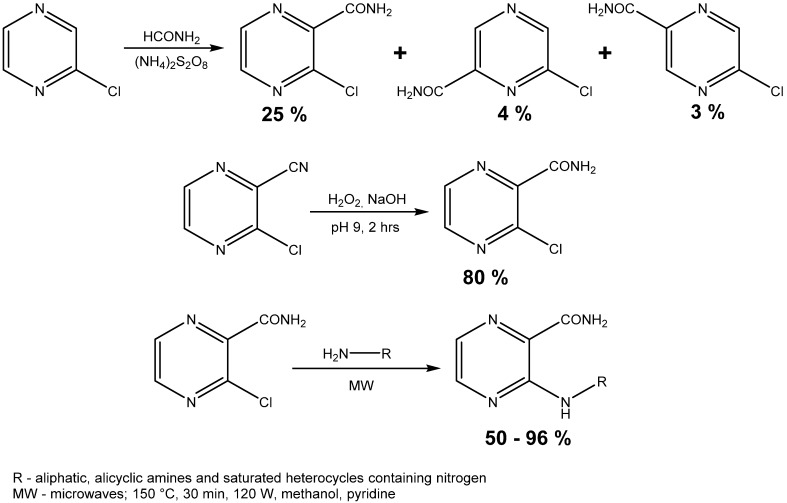
Synthesis of starting compound and microwave assisted synthesis of final compounds.

Compounds synthesized in this work were prepared according to the general procedures shown in Scheme 1. The identity and purity were proven by NMR spectra, elemental analysis and melting point.

The second method of starting compound preparation was approximately three times more efficacious than the first one comparing the yields of the reactions (80% *vs.* 25%). The melting point of 3-chloropyrazine-2-carboxamide was compared to the value stated in a previously published paper and the results were practically the same, as our measured value was in the 187.1–189.2 °C range and the reported values were 186.0–186.5 °C [33] and 188.0–190.0 °C [34]. 3-Chloropyrazine-2-carboxamide was then treated with 18 aliphatic or alicyclic amines and saturated heterocycles containing nitrogen. A series of experiments was done at the beginning to compare the efficiency of both methods—conventional and microwave-assisted heating (Table 1).

The remaining reactions were performed in microwave reactor and the conditions, which were proven experimentally, were as follows: 140 °C, 30 min, 120 W, methanol as a solvent and pyridine as a base (Scheme 1). Yields obtained from the aminodehalogenation were in a range between 50.0% and 95.8% and their variability can be explained by the steric effects of some substituents (dibutyl-, *tert*-pentyl-) that lead to worse outcomes.

This reaction yielded *N*-substituted 3-aminopyrazine-2-carboxamides (Table 2). We prepared a series consisted of 18 compounds of which 16 were novel. They were analytically characterized (NMR and IR spectra, melting point, elemental analysis) and submitted to biological screening. The obtained analytical data were fully consistent with the proposed structures. 3-(Methylamino)pyrazine-2-carboxamide was previously synthesized by Osden *et al.* (reported melting point was 200–201 °C) and by Albert *et al.* (reported melting point 198–199 °C) [33,35]. The light yellow crystalline compound we obtained melted at 200.0–201.4 °C. 3-(Cyclohexylamino)pyrazine-2-carboxamide was previously synthesized by Keir *et al.* and reported melting point was 128–129 °C [36]. Our light yellow crystalline compound melted at 129.0–130.3 °C.

**Table 1:** Table comparing the efficiency of conventional and microwave-assisted methodology showing the yields, reaction times and conditions.

Compound		Type of Synthesis	Time	Conditions	Yield
**1**	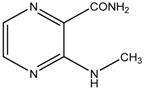	MW	30 min	140 °C, 120 W, MeOH, overpressure, 1 eq. pyridine, 2 eq. amine	61.4%
		Conventional	60 min	110 °C, toluene, 1 eq. pyridine, 2 eq. amine	53.0%
**2**	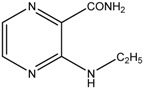	MW	30 min	140 °C, 120 W, MeOH, overpressure, 1 eq. pyridine, 2 eq. amine	78.7%
		Conventional	60 min	110 °C, toluene, 1 eq. pyridine, 2 eq. amine	46.3%
**3**	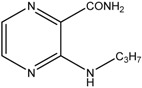	MW	30 min	140 °C, 120 W, MeOH, overpressure, 1 eq. pyridine, 2 eq. amine	87.0%
		Conventional	60 min	110 °C, toluene, 1 eq. pyridine, 2 eq. amine	56.1%
**10**	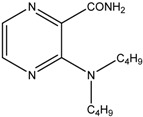	MW	30 min	140 °C, 120 W, MeOH, overpressure, 1 eq. pyridine, 2 eq. amine	67.2%
		Conventional	60 min	110 °C, toluene, 1 eq. pyridine, 2 eq. amine	0%

**Table 2:** List of prepared compounds and their lipophilicity.

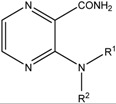				
Nr.	R^1^	R^2^	log*P*/Clog*P*	log*k*
1	CH_3_	H	−1.10/0.20	−0.4028
2	C_2_H_5_	H	−0.76/0.73	−0.2014
3	C_3_H_7_	H	−0.27/1.25	0.0038
4	C_4_H_9_	H	0.14/1.78	0.2287
5	C_5_H_11_	H	0.56/2.31	0.4563
6	*tert*-C_5_H_11_	H	0.26/1.96	0.5468
7	C_6_H_13_	H	0.98/2.84	0.6860
8	C_7_H_15_	H	1.40/3.37	0.9198
9	C_8_H_17_	H	1.81/3.90	1.1533
10	C_4_H_9_	C_4_H_9_	2.17/3.40	0.5197
11	cyclopentyl	H	0.03/1.67	0.2537
12	cyclohexyl	H	0.45/2.23	0.4523
13	cycloheptyl	H	0.87/2.79	0.6575
14	–(CH_2_)_4_–		0.01/0.34	−0.4889
15	–(CH_2_)_5_–		0.42/0.90	−0.2333
16	–(CH_2_)_2_O(CH_2_)_2_–		−0.71/−0.49	−0.6238
17	–(CH_2_)_2_NCH_3_(CH_2_)_2_–		−0.55/0.08	−0.4253
18	–(CH_2_)_2_NH(CH_2_)_2_–		−0.93/−0.50	−2.3522

### 2.2. Calculated and Experimentally Determined Lipophilicity

**Figure 1:**
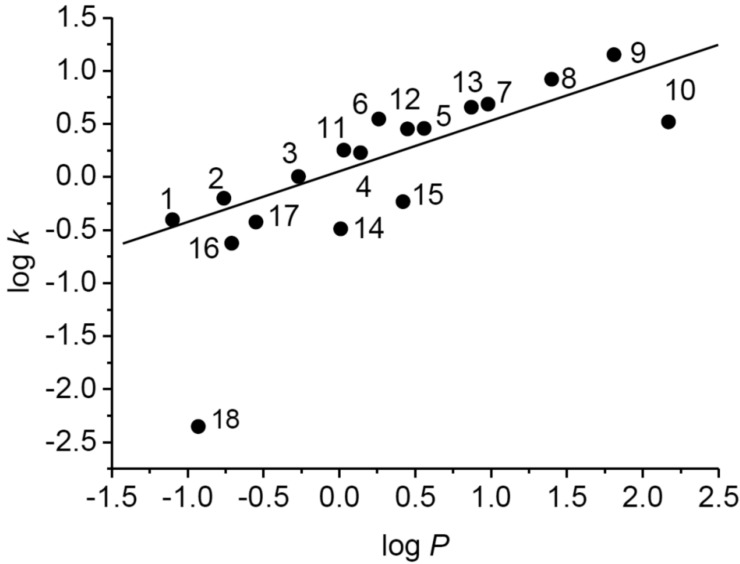
Dependence of experimentally determined log*k* on calculated log*P* values of tested compounds.

The dependence of experimentally determined log*k* on calculated log*P* values of tested compounds is shown in Figure 1. While for compounds **1**–**17** linear dependence of log*k* on log*P* was determined (*r* = 0.816), the estimated log*k* value for 3-(piperazin-1-yl)pyrazine-2-carboxamide (**18**) was considerably lower than expected. This could be connected with the presence of a secondary cyclic amine that can be very easily ionized.

### 2.3. Biological Assays

#### 2.3.1. Antimycobacterial *in Vitro* Screening

All prepared compounds were tested against the wild strain *Mycobacterium tuberculosis* H37Rv. Four substances (compounds **6**‒**9**) showed low activity against this strain with corresponding minimal inhibition concentration (MIC) values equal to 50 μg/mL. However, this activity was negligible comparing to the activity of the standard isoniazid (0.39 μg/mL) and pyrazinamide (12.5 μg/mL).

#### 2.3.2. Antifungal and Antibacterial *in Vitro* Screening

The results of antibacterial screening have shown only low activities against *Staphylococcus epidermidis*. The antibacterial activity of compounds **5**, **7** and **13** expressed by 80% inhibition concentration (IC_80_) values was lower than 250 μmol/L and did not reach the values of antibiotic standards.

Antifungal evaluation showed more interesting results. There were seven active substances and two of them (compounds **12** and **13**) showed better activity than two standards (voriconazole, fluconazole) (Table 3). The alicyclic side chain with seven carbons was more active than the 6-membered ring. The 80% inhibition of control was observed in the range from 15.62 to 31.25 μmol/L for compound **12** and in the range from 7.81 to 31.25 μmol/L for compound **13** against nearly a whole spectrum of fungi tested after 24 h of incubation. Five substances (compounds **4**, **5**, **7**, **8** and **11**) were less active than the standards and their IC_80_ values were not lower than 250 μmol/L. The activity spectrum of these compounds was also wider and did not include only one fungal strain.

**Table 3:** Results of antifungal evaluation for the compounds with alicyclic side chains and corresponding standards.

		IC_80_ [μmol/L]						
		11	12	13	AMP	VOR	NYS	FLU
**CA**	24 h	500	31.25	15.62	0.12	0.008	0.98	0.24
	48 h	>500	62.5	31.25	0.49	0.008	1.95	0.24
**CT**	24 h	>500	125	62.5	1.95	250	1.95	>500
	48 h	>500	500	500	1.95	250	3.9	>500
**CK**	24 h	250	15.62	7.81	1.95	0.98	1.95	125
	48 h	500	62.5	31.25	1.95	1.95	3.9	250
**CG**	24 h	250	15.62	7.81	0.98	250	1.95	31.25
	48 h	>500	31.25	15.62	1.95	250	3.9	250
**TA**	24 h	500	31.25	31.25	0.49	7.81	1.95	250
	48 h	>500	250	125	0.98	31.25	1.95	500
**AF**	24 h	500	15.62	7.81	1.95	0.49	1.95	>500
	48 h	>500	62,5	7.81	1.95	0.98	3.9	>500
**LC**	24 h	>500	250	62.5	7.81	250	15.62	>500
	48 h	>500	500	250	7.81	250	31.25	>500
**TM**	72 h	500	15.62	15.62	1.95	0.06	3.9	7.81
	120 h	500	15.62	15.62	1.95	0.12	7.81	125

CA: *Candida albicans*; CT: *C. tropicalis*; CK: *C. krusei*; CG: *C. glabrata*; TA: *Trichosporon asahii*; AF: *Aspergillus fumigatus*; LC: *Lichtheimia corymbifera*; TM: *Trichophyton mentagrophytes*; AMP: amphotericin B; VOR: voriconazole; NYS: nystatin; FLU: fluconazole.

It is not possible to predict structure-activity relationships for antibacterial and antifungal efficacy of studied compounds in the whole tested group due to small number of active substances and a lack of IC_80_ values better than standards. However, a saturated ring is probably important for antifungal activity and the activity also increases with increasing lipophilicity of the compounds. Unfortunately, the series is too small (only three compounds) to prove this hypothesis.

#### 2.3.3. Photosynthetic Electron Transport Inhibiting Activity Evaluation

The 3-substituted pyrazine-2-carboxamide derivatives were tested for their PET inhibiting activity. However, the PET-inhibiting activity, expressed by IC_50_ value (compound concentration in mol/L causing 50% inhibition of PET) in the investigated set, could be determined only for nine compounds. The values varied in a wide concentration range from 14.3 μmol/L (compound **9**) to 1590.0 μmol/L (compound **3**) (Table 4). The rest of compounds **1**, **2**, **10**, **14**, **15**, **16**, **17**, and **18** did not inhibit PET in spinach chloroplasts. Because all tested compounds containing heterocyclic amines as substituent in position 3 on the pyrazine ring, *i.e.*, **14**, **15**, **16**, **17**, and **18**, were found to be inactive it could be concluded that increased basicity of these compounds caused by the introduction of nitrogen atom(s) into the ring is connected with activity loss. Moreover, these substituents ultimately create a trisubstituted amine while by introduction of amino alkyl or amino cycloalkyl substituents to pyrazine-2-carboxamide disubstituted amine is formed. However, it could be stressed that trisubstituted amine is present also in compound with R = (C_4_H_9_)_2_ (**10**) which did not show PET inhibiting activity as well.

**Table 4:** IC_50_ values [μmol/L] of tested compounds related to PET inhibition in spinach chloroplasts in comparison with 3-(3,4-dichlorophenyl)-1,1-dimethylurea (DCMU) standard.

Compound	IC_50_ [μmol/L]
**3**	1590
**4**	203
**5**	38.6
**6**	480
**7**	32.9
**9**	14.3
**11**	25.8
**12**	83.0
**13**	19.3
**DCMU**	1.9

**Figure 2:**
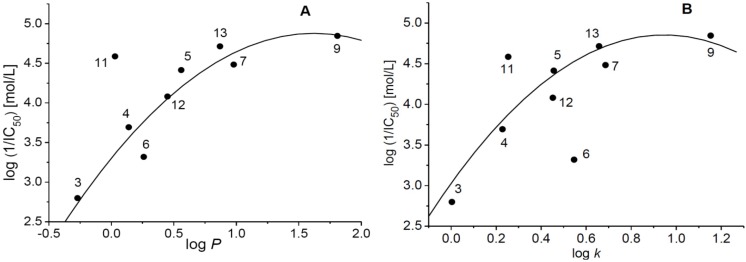
Dependence of PET-inhibiting activity on lipophilicity of studied compounds expressed as log*P* (**A**) or log*k* (**B**).

The PET-inhibiting activity was strongly affected by lipophilic properties of the compounds (Figure 2) expressed as log*P* or log*k*. The PET-inhibiting activity increased with increasing lipophilicity of the compound and the dependence of log (1/IC_50_) *vs.* log*P* showed quasi-parabolic course (Figure 2A). The only exception was compound **11** (R = cyclopentyl with log*P* = 0.03) activity of which was considerably higher (IC_50_ = 25.8 μmol/L) than that of compound **4** with log*P* = 0.14 (IC_50_ = 203 μmol/L). Similarly, quasi-parabolic course was also observed for the dependence of PET inhibiting activity on log*k*. However, with respect to the quasi-parabolic course of this dependence, compound **11** (log*k* = 0.2537) showed higher and compound **6** (log*k* = 0.5468) lower PET inhibiting activity (Figure 2B).

The dependence of log (1/IC_50_) on log*P* can be expressed by following equation after excluding the 3-(cyclopentylamino)pyrazine-2-carboxamide (**11**):

log(1/IC_50_) = 3.302 (±0.137) + 1.943 (±0.366) log*P* − 0.599 (±0.211) (log*P*)^2^ *r* = 0.955, s = 0.253, F = 25.79, *n* = 8 (1)

On the other hand, after excluding the 3-(*tert*-pentylamino)pyrazine-2-carboxamide (6) the dependence of log (1/IC_50_) on log*k* can be expressed by following equation:

log (1/IC_50_) = 3.028 (±0.329) + 0.386 (±0.126) log*k* − 2.040 (±1.028) (log*k*)^2^ *r* = 0.881, s = 0.378, F = 8.68, *n* = 8 (2)

The obtained results indicated that IC_50_ could not be determined for compounds with too low lipophilicity, namely **1** (log*P* = −1.3), **2** (log*P* = −0.76).

As mentioned above, the ineffectiveness of compound **10** **(**R = (C_4_H_9_)_2_) as well as compounds **14**‒**18** with heterocyclic substituents could be connected mainly with the absence of a secondary amine fragment (-NH-) bounded to the pyrazine ring. Consequently, possible interactions of these compounds with constituents of the photosynthetic apparatus are limited. On the other side, a 2-carboxamino substituent CONH_2_ on the pyrazine ring plays an important role in the interactions of the tested pyrazine derivatives with constituents of the photosynthetic apparatus. Moreover, increasing length of the alkyl substituent contributes not only leads to better transport of the compound to its site of action but incorporation of longer alkyl chains into the thylakoid membrane that results in membrane damage and consecutive increase of PET inhibiting activity. Similar results were also obtained with 2-alkylthio-4-pyridinecarbothioamides [26].

Artificial electron donors could be used to specify the site of inhibitory action of PET inhibitors in the photosynthetic apparatus because they can restore PET activity in chloroplasts of which was previously inhibited. For example, 2,5-diphenylcarbazide (DPC) can supply electrons in the site of Z**^•^**/D**^•^** intermediate which is situated on the donor side of PS2 [37]. Consequently, if PET was inhibited in the section between the oxygen evolving complex and Z**^•^**/D**^•^** intermediate, DPC can restore PET between the core of PS2 (P680) and secondary quinone acceptor Q_B_ which is situated on the acceptor side of PS2 and 2,6-dichlorophenol-indophenol (DCPIP) photoreduction occurs again. However, if the site of PET inhibitor is located between P680 and Q_B_, photoreduction of DCPIP (an artificial electron acceptor used for monitoring the PET through PS2 from H_2_O to Q_B_) remains inhibited. Activity of chloroplasts, which was restricted by studied pyrazine-2-carboxamides, was practically completely restored after DPC addition. Thus, it can be concluded that the site of action of tested compounds in the electron transport chain is situated exclusively on the donor side of PS2, between oxygen evolving complex and Z**^•^**/D**^•^** intermediate, while the section of the electron transport chain on the acceptor side of PS2 between P680 and Q_B_ is not damaged. Similar results were also obtained with *N*-substituted 5-amino-6-methylpyrazine-2,3-dicarbonitriles [23], 2-benzylsulphanylbenzimidazoles [38] and ring-substituted 1-hydroxynaphthalene-2-carboxanilides [39]. On the other hand, addition of DPC restored PET in chloroplasts, which was inhibited by *N-*benzylpyrazine-2-carboxamides only to 77%–88% indicating that the site of PET inhibition is situated not only on the donor but also on the acceptor side of PS2 [22]. Similar sites of action on both sides of PS2 were determined previously for 5-*tert*-butyl-*N*-(3-hydroxy-4-chlorophenyl)-pyrazine-2-carboxamide and 5-*tert*-butyl-6-chloro-*N*-(3-fluorophenyl)pyrazine-2-carboxamide [24].

**Figure 3:**
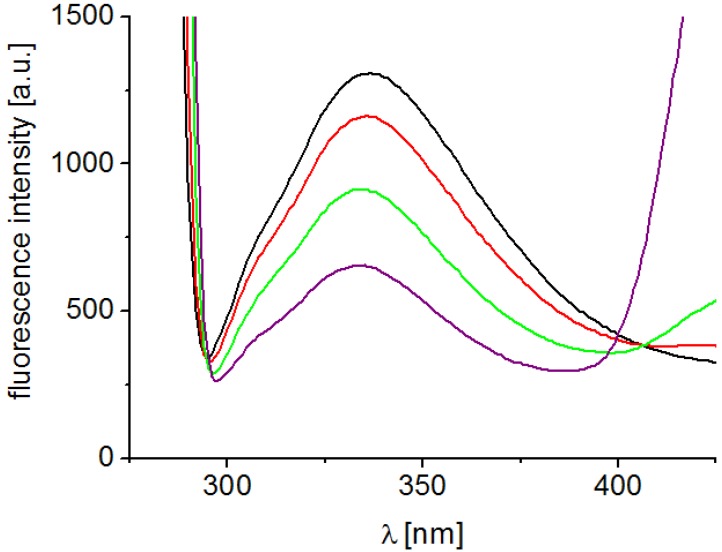
Fluorescence emission spectra of aromatic amino acids in suspension of spinach chloroplasts without and with compound **7** (c = 0, 6, 24 and 48 μmol/L; the curves from top to bottom); excitation wave length λ = 275 nm; chlorophyll concentration 10 mg/L.

Interaction of substituted pyrazine-2-carboxamides with residues of aromatic amino acids (AAA), mainly tryptophan and tyrosine occurring in photosynthetic proteins situated mainly in PS2, which was documented by the quenching of AAA fluorescence at 334 nm contributed to PET inhibition. Figure 3 presents fluorescence emission spectra of AAA of untreated spinach chloroplasts and of chloroplasts treated with increasing concentrations of compound **7**. As shown in Figure 3, the quenching of the fluorescence of aromatic amino acids at 334 nm increased with increasing concentration of pyrazine derivative. This finding is in accordance with the results obtained in the above mentioned study of PET inhibition in spinach chloroplasts by tested pyrazine-2-carboxamides using artificial electron acceptor DCPIP.

The quenching of the fluorescence of aromatic amino acids in the presence of 5-bromo- and 3,5-dibromo-2-hydroxy-*N*-phenylbenzamides and ring-substituted 2-hydroxynaphthalene-1-carboxanilides was observed previously [40,41].

## 3. Experimental

### 3.1. General

All chemicals were reagent or higher grade of purity and were purchased from Sigma-Aldrich (Steinheim, Germany) or Fluorochem Ltd. (Hadfield, Derbyshire, UK), unless stated otherwise.

The starting compound was prepared according to proven conventional organic synthesis methodology. The final aminodehalogenation reaction was performed in a CEM Discover microwave reactor with a focused field (CEM Corporation, Matthews, NC, USA) connected to an Explorer 24 autosampler (CEM Corporation) and this equipment was running under CEM’s Synergy^TM^ software for setting and monitoring the conditions of reactions. The temperature of the reaction mixture was monitored by internal infrared sensor. The progress of the reaction was checked by Thin Layer Chromatography (TLC) (Alugram^®^ Sil G/UV_254_, Machery-Nagel, Postfach, Germany) with UV detection using wavelength 254 nm.

All obtained products were purified by crystallization or by preparative flash chromatograph CombiFlash^®^ Rf (Teledyne Isco Inc., Lincoln, NE, USA). The type of elution was gradient, using the mixture of hexane (LachNer, Neratovice, Czech Republic) and ethyl acetate (Penta, Prague, Czech Republic) as mobile phase. Silica gel (0.040–0.063 nm, Merck, Darmstadt, Germany) was used as the stationary phase.

NMR spectra were taken with spectrometers Varian Mercury-VxBB 300 with frequencies 299.95 MHz for ^1^H and 75.43 MHz for ^13^C or Varian VNMR S500 (499.87 MHz for ^1^H and 125.71 MHz for ^13^C) (Varian Corporation, Palo Alto, CA, USA). Chemical shifts were reported in ppm (δ) and were applied indirectly to tetramethylsilane as a signal of solvent (2.49 for ^1^H and 39.7 for ^13^C in DMSO-*d*_6_). Infrared spectra were recorded with spectrometer FT-IR Nicolet 6700 (Thermo Scientific, Waltham, MA, USA) using attenuated total reflectance (ATR) methodology. Elemental analyses were measured with EA 1110 CHNS Analyzer (Fisons Instruments S. p. A., Carlo Erba, Milano, Italy). Melting points were assessed by SMP3 Stuart Scientific (Bibby Sterling Ltd., Staffordshire, UK) and were uncorrected. Log*P* and Clog*P* were calculated with PC program CS ChemBioDraw Ultra 13.0 (CambridgeSoft, Cambridge, MA, USA).

### 3.2. Synthesis of Starting Compound and Final Products

The starting compound 3-chloropyrazine-2-carboxamide was synthesized using two published procedures. The first method was classified as less effective and was based on the homolytic amidation of 2-chloropyrazine. Thus, 2-chloropyrazine (0.17 mol) was dissolved in formamide (3.7 mol), heated to 90 °C and ammonium peroxodisulphate (0.18 mol) was added portionwise over one hour period. This mixture reacted for another one hour at 90 °C and then it was left to stand for 24 h at laboratory temperature. Dilution with 100 mL of water was followed by filtration and this filtrate was extracted continuously with chloroform for 16 h [34,42]. The mixture of three positional isomers was separated by flash chromatography using silica gel as stationary phase. The second process used 3-chloropyrazine-2-carbonitrile, which was submitted to partial hydrolysis of the nitrile group. The powdered carbonitrile (0.104 mol) was added little by little into the reaction mixture of concentrated hydrogen peroxide (0.95 mol) and water (195 mL) heated to 50 °C. The pH was adjusted and regulated around a value of 9 using an 8% solution of sodium hydroxide and the temperature of the reaction was regulated between 55 and 60 °C. The reaction was stopped after 2.5 h and was cooled to 5 °C. Newly-emerged crystals were removed by suction and recrystallized from ethanol [42].

The starting compound (1.27 mmol) was treated with 18 aliphatic amines, alicyclic amines or saturated heterocycles containing at least one nitrogen atom (2.54 mmol). Four reactions were completed by conventional heating methods. The conditions were 110 °C, toluene as a solvent and pyridine (1.27 mmol) as a base. The reaction time was set to one hour. Then the reactions were completed using the microwave reactor with focused field and conditions used for syntheses were 140 °C, 30 min, 120 W, methanol used as a solvent and pyridine (1.27 mmol) as a base. They were set experimentally with respect to prior experience. The progress of reaction was monitored with TLC in system hexane/ethyl acetate (1:1). Then the mixture was separated by flash column chromatograph using gradient elution. Mobile phases were hexane and ethyl acetate again.

### 3.3. Analytical Data of the Prepared Compounds

*3-(Methylamino)pyrazine-2-carboxamide* (**1**). Light yellow crystalline solid. Yield 61.4%; m.p. 200.0–201.4 °C (described in the literature 200–201 °C [35] or 198–199 °C [33]); IR (ATR-Ge, cm^−1^): 3413_m_ (-NH-), 3293_m_, 3179_m_ (-CO**NH_2_**), 1686_vs_ (-**CO**NH_2_), 1589_s_, 1513_s_, 1436_m_, 1414_s_, 1194_vs_ (pyrazine ring); ^1^H-NMR (300 MHz, DMSO-*d*_6_) δ 8.65–8.55 (1H, m, NH), 8.23 (1H, d, *J* = 2.3 Hz, Ar), 8.11 (1H, bs, NH_2_), 7.72 (1H, d, *J* = 2.3 Hz, Ar), 7.63 (1H, bs, NH_2_), 2.90 (3H, d, *J* = 5.3 Hz, CH_3_); ^13^C-NMR (75 MHz, DMSO-*d*_6_) δ 168.9, 155.1, 146.6, 129.5, 126.9, 27.3; Elemental analysis: calc. for C_6_H_8_N_4_O (MW 152.15): 47.36% C, 5.30% H, 36.82% N; found 47.43% C, 5.45% H, 36.68% N.

*3-(Ethylamino)pyrazine-2-carboxamide* (**2**). Light yellow crystalline solid. Yield 78.7%; m.p. 117.4–118.5 °C; IR (ATR-Ge, cm^−1^): 3381_m_ (-NH-), 3294_m_, 3209_m_ (-CO**NH_2_**), 1683_vs_ (-**CO**NH_2_), 1582_s_, 1533_m_, 1500_vs_, 1419_m_, 1192_vs_ (pyrazine ring); ^1^H-NMR (300 MHz, DMSO-*d*_6_) δ 8.68 (1H, t, *J* = 4.7 Hz, NH), 8.22 (1H, d, *J* = 2.3 Hz, Ar), 8.11 (1H, bs, NH_2_), 7.72 (1H, d, *J* = 2.3 Hz, Ar), 7.64 (1H, bs, NH_2_), 3.47–3.32 (2H, m, NCH_2_), 1.14 (3H, t, *J* = 7.0 Hz, CH_3_); ^13^C-NMR (75 MHz, DMSO-*d*_6_) δ 169.0, 154.4, 146.6, 129.6, 126.5, 34.8, 14.8; Elemental analysis: calc. for C_7_H_10_N_4_O (MW 166.18): 50.59% C, 6.07% H, 33.71% N; found 50.66% C, 5.98% H, 33.51% N.

*3-(Propylamino)pyrazine-2-carboxamide* (**3**). Yellow crystalline solid. Yield 87.0%; m.p. 93.9–94.9 °C; IR (ATR-Ge, cm^−1^): 3402_m_ (-NH-), 3337_m_, 3186_m_ (-CO**NH_2_**), 1671_vs_ (-**CO**NH_2_), 1578_s_, 1533_m_, 1514_vs_, 1423_s_, 1189_vs_ (pyrazine ring); ^1^H-NMR (300 MHz, DMSO-*d*_6_) δ 8.77 (1H, t, *J* = 6.5 Hz, NH), 8.21 (1H, d, *J* = 2.3 Hz, Ar), 8.12 (1H, bs, NH_2_), 7.72 (1H, d, *J* = 2.3 Hz, Ar), 7.64 (1H, bs, NH_2_), 3.34 (2H, q, *J* = 6.5 Hz, NCH_2_), 1.63–1.46 (2H, m, CH_2_), 0.89 (3H, t, *J* = 6.5 Hz, CH_3_); ^13^C-NMR (75 MHz, DMSO-*d*_6_) δ 169.1, 154.6, 146.6, 129.6, 126.5, 41.7, 22.2, 11.6; Elemental analysis: calc. for C_8_H_12_N_4_O (MW 180.21): 53.32% C, 6.71% H, 31.09% N; found 53.45% C, 6.79% H, 30.93% N.

*3-(Butylamino)pyrazine-2-carboxamide* (**4**). Yellow crystalline solid. Yield 92.3%; m.p. 83.1–84.4 °C; IR (ATR-Ge, cm^−1^): 3409_m_ (-NH-), 3326_m_, 3177_m_ (-CO**NH_2_**), 1671_vs_ (-**CO**NH_2_), 1578_s_, 1533_m_, 1518_vs_, 1423_s_, 1187_vs_ (pyrazine ring); ^1^H-NMR (300 MHz, DMSO-*d*_6_) δ 8.75 (1H, t, *J* = 6.6 Hz, NH), 8.22 (1H, d, *J* = 2.3 Hz, Ar), 8.12 (1H, bs, NH_2_), 7.73 (1H, d, *J* = 2.3 Hz, Ar), 7.64 (1H, bs, NH_2_), 3.38 (2H, q, *J* = 6.6 Hz, NCH_2_), 1.59–1.46 (2H, m, CH_2_), 1.41–1.25 (2H, m, CH_2_), 0.88 (3H, t, *J* = 6.6 Hz, CH_3_); ^13^C-NMR (75 MHz, DMSO-*d*_6_) δ 169.1, 154.6, 146.6, 129.6, 126.5, 40.5, 31.1, 19.9, 13.9; Elemental analysis: calc. for C_9_H_14_N_4_O (MW 194.23): 55.65% C, 7.27% H, 28.85% N; found 55.77% C, 7.27% H, 28.93% N.

*3-(Pentylamino)pyrazine-2-carboxamide* (**5**). Light yellow crystalline solid. Yield 92.8%; m.p. 105.0–106.3 °C; IR (ATR-Ge, cm^−1^): 3379_m_ (-NH-), 3321_m_, 3145_m_ (-CO**NH_2_**), 1663_vs_ (-**CO**NH_2_), 1587_s_, 1531_m_, 1509_vs_, 1418_s_, 1185_vs_ (pyrazine ring); ^1^H-NMR (300 MHz, DMSO-*d*_6_) δ 8.76 (1H, t, *J* = 6.5 Hz, NH), 8.22 (1H, d, *J* = 2.3 Hz, Ar), 8.12 (1H, bs, NH_2_), 7.73 (1H, d, *J* = 2.3 Hz, Ar), 7.64 (1H, bs, NH_2_), 3.38 (2H, q, *J* = 6.5 Hz, NCH_2_), 1.62–1.46 (2H, m, CH_2_), 1.37–1.19 (4H, m, CH_2_), 0.85 (3H, t, *J* = 6.5 Hz, CH_3_); ^13^C-NMR (75 MHz, DMSO-*d*_6_) δ 169.1, 154.6, 146.6, 129.6, 126.4, 40.5, 28.9, 28.7, 22.1, 14.1; Elemental analysis: calc. for C_10_H_16_N_4_O (MW 208.26): 57.67% C, 7.74% H, 26.90% N; found 57.63% C, 7.67% H, 27.06% N.

*3-(tert-Pentylamino)pyrazine-2-carboxamide* (**6**). Yellow crystalline solid. Yield 51.3%; m.p. 65.0–66.7 °C; IR (ATR-Ge, cm^−1^): 3396_m_ (-NH-), 3265_m_, 3185_m_ (-CO**NH_2_**), 1668_vs_ (-**CO**NH_2_), 1581_vs_, 1504_vs_, 1415_s_, 1183_vs_ (pyrazine ring); ^1^H-NMR (300 MHz, CDCl_3_) δ 8.65 (1H, bs, NH), 8.13 (1H, d, *J* = 2.3 Hz, Ar), 7.74 (1H, bs, NH_2_), 7.61 (1H, d, *J* = 2.3 Hz, Ar), 5.56 (1H, bs, NH_2_), 1.86 (2H, q, *J* = 7.6 Hz, CH_2_), 1.42 (6H, s, CH_3_), 0.87 (3H, t, *J* = 7.6 Hz, CH_3_); ^13^C-NMR (75 MHz, CDCl_3_) δ 169.7, 154.9, 146.3, 128.9, 125.5, 54.2, 33.0, 26.4, 8.4; Elemental analysis: calc. for C_10_H_16_N_4_O (MW 208.26): 57.67% C, 7.74% H, 26.90% N; found 57.80% C, 7.89% H, 26.70% N.

*3-(Hexylamino)pyrazine-2-carboxamide* (**7**). Light yellow crystalline solid. Yield 93.6%; m.p. 64.3–65.6 °C; IR (ATR-Ge, cm^−1^): 3404_m_ (-NH-), 3314_m_, 3192_m_ (-CO**NH_2_**), 1659_vs_ (-**CO**NH_2_), 1587_vs_, 1536_m_, 1509_vs_, 1424_m_, 1190_vs_ (pyrazine ring); ^1^H-NMR (300 MHz, DMSO-*d*_6_) δ 8.76 (1H, t, *J* = 6.2 Hz, NH), 8.22 (1H, d, *J* = 2.3 Hz, Ar), 8.12 (1H, bs, NH_2_), 7.73 (1H, d, *J* = 2.3 Hz, Ar), 7.64 (1H, bs, NH_2_), 3.38 (2H, q, *J* = 6.2 Hz, NCH_2_), 1.60–1.44 (2H, m, CH_2_), 1.39–1.16 (6H, m, CH_2_), 0.84 (3H, t, *J* = 6.2 Hz, CH_3_); ^13^C-NMR (75 MHz, DMSO-*d*_6_) δ 169.1, 154.6, 146.6, 129.6, 126.4, 40.5, 31.2, 28.9, 26.4, 22.2, 14.1; Elemental analysis: calc. for C_11_H_18_N_4_O (MW 222.29): 59.44% C, 8.16% H, 25.20% N; found 59.41% C, 8.24% H, 25.10% N.

*3-(Heptylamino)pyrazine-2-carboxamide* (**8**). Yellow crystalline solid. Yield 91.5%; m.p. 66.0–67.3 °C; IR (ATR-Ge, cm^−1^): 3407_m_ (-NH-), 3316_m_, 3176_m_ (-CO**NH_2_**), 1674_vs_ (-**CO**NH_2_), 1580_vs_, 1532_m_, 1514_vs_, 1421_m_, 1184_s_ (pyrazine ring); ^1^H-NMR (300 MHz, DMSO-*d*_6_) δ 8.76 (1H, t, *J* = 6.3 Hz, NH), 8.22 (1H, d, *J* = 2.3 Hz, Ar), 8.12 (1H, bs, NH_2_), 7.72 (1H, d, *J* = 2.3 Hz, Ar), 7.64 (1H, bs, NH_2_), 3.38 (2H, q, *J* = 6.3 Hz, NCH_2_), 1.60–1.45 (2H, m, CH_2_), 1.37–1.15 (8H, m, CH_2_), 0.83 (3H, t, *J* = 6.3 Hz, CH_3_); ^13^C-NMR (75 MHz, DMSO-*d*_6_) δ 169.1, 154.6, 146.6, 129.6, 126.4, 40.5, 31.4, 29.0, 28.6, 26.7, 22.2, 14.1; Elemental analysis: calc. for C_12_H_20_N_4_O (MW 236.31): 60.99% C, 8.53% H, 23.71% N; found 61.12% C, 8.57% H, 23.64% N.

*3-(Octylamino)pyrazine-2-carboxamide* (**9**). Light yellow crystalline solid. Yield 95.8%; m.p. 67.2–68.3 °C; IR (ATR-Ge, cm^−1^): 3407_m_ (-NH-), 3321_m_, 3211_m_ (-CO**NH_2_**), 1664_vs_ (-**CO**NH_2_), 1594_vs_, 1539_m_, 1510_vs_, 1423_m_, 1197_vs_ (pyrazine ring); ^1^H-NMR (300 MHz, DMSO-*d*_6_) δ 8.76 (1H, t, *J* = 6.4 Hz, NH), 8.22 (1H, d, *J* = 2.3 Hz, Ar), 8.11 (1H, bs, NH_2_), 7.73 (1H, d, *J* = 2.3 Hz, Ar), 7.64 (1H, bs, NH_2_), 3.38 (2H, q, *J* = 6.4 Hz, NCH_2_), 1.61–1.45 (2H, m, CH_2_), 1.37–1.14 (10H, m, CH_2_), 0.83 (3H, t, *J* = 6.4 Hz, CH_3_); ^13^C-NMR (75 MHz, DMSO-*d*_6_) δ 169.1, 154.6, 146.6, 129.6, 126.5, 40.5, 31.4, 29.0, 28.9, 28.8, 26.7, 22.3, 14.1; Elemental analysis: calc. for C_13_H_22_N_4_O (MW 250.34): 62.37% C, 8.86% H, 22.38% N; found 62.42% C, 8.81% H, 22.25% N.

*3-(Dibutylamino)pyrazine-2-carboxamide* (**10**). Light yellow crystalline solid. Yield 67.2%; m.p. 129.3–130.8 °C; IR (ATR-Ge, cm^−1^): 3372_m_, 3197_m_ (-CO**NH_2_**), 1647_vs_ (-**CO**NH_2_), 1616_m_, 1558_m_, 1507_m_, 1457_m_, 1179_s_ (pyrazine ring); ^1^H-NMR (300 MHz, DMSO-*d*_6_) δ 8.08 (1H, d, *J* = 2.3 Hz, Ar), 7.96 (1H, bs, NH_2_), 7.73 (1H, d, *J* = 2.3 Hz, Ar), 7.52 (1H, bs, NH_2_), 3.40 (4H, q, *J* = 7.5 Hz, NCH_2_), 1.56–1.42 (4H, m, CH_2_), 1.31–1.13 (4H, m, CH_2_), 0.85 (6H, t, *J* = 7.5 Hz, CH_3_); ^13^C-NMR (75 MHz, DMSO-*d*_6_) δ 169.5, 151.7, 141.8, 135.5, 129.7, 48.8, 29.3, 19.8, 14.0; Elemental analysis: calc. for C_13_H_22_N_4_O (MW 250.34): 62.37% C, 8.86% H, 22.38% N; found 62.27% C, 8.66% H, 22.32% N.

*3-(Cyclopentylamino)pyrazine-2-carboxamide* (**11**). Yellow crystalline solid. Yield 90.5%; m.p. 107.8–108.8 °C; IR (ATR-Ge, cm^−1^): 3399_m_ (-NH-), 3285_m_ (-CO**NH_2_**), 1655_vs_ (-**CO**NH_2_), 1580_vs_, 1525_m_, 1505_vs_, 1419_m_, 1172_vs_ (pyrazine ring); ^1^H-NMR (300 MHz, DMSO-*d*_6_) δ 8.79 (1H, d, *J* = 7.0 Hz, NH), 8.23 (1H, d, *J* = 2.3 Hz, Ar), 8.12 (1H, bs, NH_2_), 7.73 (1H, d, *J* = 2.3 Hz, Ar), 7.64 (1H, bs, NH_2_), 4.33–4.12 (1H, m, NCH), 2.05–1.88 (2H, m, CH_2_), 1.75–1.49 (4H, m, CH_2_), 1.49–1.33 (2H, m, CH_2_); ^13^C-NMR (75 MHz, DMSO-*d*_6_) δ 169.1, 154.2, 146.6, 129.7, 126.4, 51.5, 32.9, 23.5; Elemental analysis: calc. for C_10_H_14_N_4_O (MW 206.24): 58.24% C, 6.84% H, 27.17% N; found 58.23% C, 6.85% H, 26.98% N.

*3-(Cyclohexylamino)pyrazine-2-carboxamide* (**12**). Light yellow crystalline solid. Yield 92.5%; m.p. 129.0–130.3 °C (described in the literature 128–129 °C [36]); IR (ATR-Ge, cm^−1^): 3455_m_ (-NH-), 3254_m_ (-CO**NH_2_**), 1683_vs_ (-**CO**NH_2_), 1583_s_, 1518_vs_, 1473_m_, 1418_m_, 1182_s_ (pyrazine ring); ^1^H-NMR (300 MHz, DMSO-*d*_6_) δ 8.78 (1H, d, *J* = 7.6 Hz, NH), 8.21 (1H, d, *J* = 2.3 Hz, Ar), 8.12 (1H, bs, NH_2_), 7.72 (1H, d, *J* = 2.3 Hz, Ar), 7.64 (1H, bs, NH_2_), 3.96–3.80 (1H, m, NCH), 1.96–1.82 (2H, m, CH_2_), 1.73–1.60 (2H, m, CH_2_), 1.60–1.47 (1H, m, CH_2_), 1.43–1.14 (5H, m, CH_2_); ^13^C-NMR (75 MHz, DMSO-*d*_6_) δ 169.1, 153.8, 146.6, 129.6, 126.2, 48.0, 32.4, 25.5, 24.4; Elemental analysis: calc. for C_11_H_16_N_4_O (MW 220.27): 59.98% C, 7.32% H, 25.44% N; found 59.78% C, 7.38% H, 25.33% N.

*3-(Cycloheptylamino)pyrazine-2-carboxamide* (**13**). Light yellow crystalline solid. Yield 87.4%; m.p. 102.7–104.1 °C; IR (ATR-Ge, cm^−1^): 3450_m_ (-NH-), 3293_m_, 3180_m_ (-CO**NH_2_**), 1674_vs_ (-**CO**NH_2_), 1581_vs_, 1506_vs_, 1465_m_, 1416_s_, 1177_vs_ (pyrazine ring); ^1^H-NMR (300 MHz, DMSO-*d*_6_) δ 8.84 (1H, d, *J* = 7.6 Hz, NH), 8.22 (1H, d, *J* = 2.3 Hz, Ar), 8.12 (1H, bs, NH_2_), 7.71 (1H, d, *J* = 2.3 Hz, Ar), 7.64 (1H, bs, NH_2_), 4.15–3.99 (1H, m, NCH), 1.95–1.79 (2H, m, CH_2_), 1.65–1.37 (10H, m, CH_2_); ^13^C-NMR (75 MHz, DMSO-*d*_6_) δ 169.1, 153.7, 146.6, 129.5, 126.3, 50.2, 34.2, 27.7, 23.7; Elemental analysis: calc. for C_12_H_18_N_4_O (MW 234.30): 61.52% C, 7.74% H, 23.91% N; found 61.50% C, 7.94% H, 24.05% N.

*3-(Pyrrolidin-1-ylamino)pyrazine-2-carboxamide* (**14**). Yellow crystalline solid. Yield 82.7%; m.p. 179.6–180.0 °C; IR (ATR-Ge, cm^−1^): 3384_m_, 3189_m_ (-CO**NH_2_**), 1638_vs_ (-**CO**NH_2_), 1551_s_, 1517_s_, 1462_s_, 1437_s_, 1191_s_ (pyrazine ring); ^1^H-NMR (300 MHz, CDCl_3_) δ 8.14 (1H, d, *J* = 2.3 Hz, Ar), 7.75 (1H, d, *J* = 2.3 Hz, Ar), 7.18 (1H, bs, NH_2_), 5.84 (1H, bs, NH_2_), 3.54–3.39 (4H, m, NCH_2_), 2.00–1.85 (4H, m, CH_2_); ^13^C-NMR (75 MHz, CDCl_3_) δ 168.8, 151.5, 143.6, 129.4, 129.2, 49.5, 25.4; Elemental analysis: calc. for C_9_H_12_N_4_O (MW 192.22): 56.24% C, 6.29% H, 29.15% N; found 56.16% C, 6.23% H, 29.11% N.

*3-(Piperidin-1-ylamino)pyrazine-2-carboxamide* (**15**). Light yellow crystalline solid. Yield 93.0%; m.p. 126.4–127.7 °C; IR (ATR-Ge, cm^−1^): 3385_m_, 3183_m_ (-CO**NH_2_**), 1646_vs_ (-**CO**NH_2_), 1552_s_, 1518_s_, 1484_vs_, 1440_vs_, 1183_vs_ (pyrazine ring); ^1^H-NMR (300 MHz, DMSO-*d*_6_) δ 8.13 (1H, d, *J* = 2.1 Hz, Ar), 7.92 (1H, bs, NH_2_), 7.82 (1H, d, *J* = 2.3 Hz, Ar), 7.51 (1H, bs, NH_2_), 3.45–3.33 (4H, m, NCH_2_), 1.64–1.42 (6H, m, CH_2_); ^13^C-NMR (75 MHz, DMSO-*d*_6_) δ 168.9, 153.0, 142.2, 135.9, 131.1, 48.2, 25.4, 24.1; Elemental analysis: calc. for C_10_H_14_N_4_O (MW 206.24): 58.24% C, 6.84% H, 27.17% N; found 58.29% C, 6.77% H, 27.37% N.

*3-(Morpholin-4-yl)pyrazine-2-carboxamide* (**16**). Light yellow crystalline solid. Yield 91.3%; m.p. 146.1–147.5 °C; IR (ATR-Ge, cm^−1^): 3330_m_, 3184_m_ (-CO**NH_2_**), 1655_vs_ (-**CO**NH_2_), 1609_m_, 1554_s_, 1519_m_, 1484_vs_, 1436_s_, 1109_vs_ (pyrazine ring); ^1^H-NMR (300 MHz, CDCl_3_) δ 8.20 (1H, d, *J* = 2.3 Hz, Ar), 7.91 (1H, d, *J* = 2.3 Hz, Ar), 7.49 (1H, bs, NH_2_), 5.87 (1H, bs, NH_2_), 3.82 (4H, t, *J* = 4.7 Hz, OCH_2_), 3.54 (4H, t, *J* = 4.7 Hz, NCH_2_); ^13^C-NMR (75 MHz, CDCl_3_) δ 167.8, 154.4, 143.6, 132.3, 131.3, 66.7, 49.0; Elemental analysis: calc. for C_9_H_12_N_4_O_2_ (MW 208.22): 51.92% C, 5.81% H, 26.91% N; found 51.80% C, 5.91% H, 26.83% N.

*3-(4-Methylpiperazin-1-yl)pyrazine-2-carboxamide* (**17**). Sandy yellow crystalline solid. Yield 50.0%; m.p. 168.8–170.5 °C (decomp.); IR (ATR-Ge, cm^−1^): 3374_m_, 3190_m_ (-CO**NH_2_**), 1647_vs_ (-**CO**NH_2_), 1552_s_, 1519_m_, 1492_vs_, 1440_s_, 1186_s_ (pyrazine ring); ^1^H-NMR (300 MHz, DMSO-*d*_6_) δ 8.16 (1H, d, *J* = 2.3 Hz, Ar), 7.97 (1H, bs, NH_2_), 7.88 (1H, d, *J* = 2.3 Hz, Ar), 7.56 (1H, bs, NH_2_), 3.42 (4H, t, *J* = 5.0 Hz, NCH_2_), 2.35 (4H, t, *J* = 5.0 Hz, NCH_2_), 2.18 (3H, s, CH_3_); ^13^C-NMR (75 MHz, DMSO-*d*_6_) δ 168.8, 152.8, 142.3, 136.1, 131.8, 54.5, 47.0, 46.0; Elemental analysis: calc. for C_10_H_15_N_5_O (MW 221.26): 54.28% C, 6.83% H, 31.65% N; found 54.19% C, 6.84% H, 31.48% N.

*3-(Piperazin-1-yl)pyrazine-2-carboxamide* (**18**). Yellow crystalline solid. Yield 65.8%; m.p. decomp.; IR (ATR-Ge, cm^−1^): 3389_m_ (-NH-), 3293_m_, 3179_m_ (-CO**NH_2_**), 1678_vs_ (-**CO**NH_2_), 1593_s_, 1559_s_, 1527_m_, 1464_s_, 1433_s_, 1159_s_ (pyrazine ring); ^1^H-NMR (300 MHz, DMSO-*d*_6_) δ 9.75 (1H, bs, NH), 8.26 (1H, bs, Ar), 8.08 (1H, bs, NH_2_), 8.02 (1H, bs, Ar), 7.67 (1H, bs, NH_2_), 3.68–3.59 (4H, m, NCH_2_), 3.16–3.07 (4H, m, NCH_2_); ^13^C-NMR (75 MHz, DMSO-*d*_6_) δ 168.2, 152.6, 142.8, 136.0, 133.4, 44.5, 42.5; Elemental analysis: calc. for C_9_H_13_N_5_O (MW 207.23): 52.16% C, 6.32% H, 33.79% N; found 52.19% C, 6.31% H, 33.59% N.

### 3.4. Lipophilicity HPLC Determination and Calculations

Experimental lipophilicity parameter log*k* was ascertained using an Agilent Technologies 1200 SL liquid chromatography HPLC system with a SL G1315C Diode-array Detector, chromatographic pre-column ZORBAX XDB-C18 5 μm, 4 × 4 mm, Part No. 7995118-504 and column ZORBAX Eclipse XDB-C18 5 μm, 4.6 × 250 mm, Part No. 7995118-585 (Agilent Technologies Inc., Colorado Springs, CO, USA) were used. The separation process was controlled by Agilent ChemStation, version B.04.02 extended by spectral module (Agilent Technologies Inc.). A solution of MeOH (HPLC grade, 70%) and H_2_O (HPLC-Milli-Q Grade, 30%) was used as mobile phase. The total flow of the column was 1.0 mL/min, injection 20 μL, column temperature 30 °C. 210 nm as detection wavelength and 270 nm as monitor wavelength were chosen. The KI methanol solution was used for the dead time (T_D_) determination. Retention times (T_R_) of synthesized compounds were measured in minutes. The capacity factors *k* were calculated using Microsoft Excel according to formula *k* = (T_R_− T_D_)/T_D_, where T_R_ is the retention time of the solute and T_D_ denotes the dead time obtained via an unretained analyte. Log*k*, calculated from the capacity factor *k*, is used as the lipophilicity index converted to log*P* scale.

### 3.5. Biological Assays

#### 3.5.1. Antimycobacterial *in Vitro* Screening

Mycobacterial screening was performed against *M. tuberculosis* H37Rv CNCTC My 331/88 (Czech National Collection of Type Cultures, National Institute of Public Health, Prague, Czech Republic) using isoniazid and pyrazinamide (Sigma-Aldrich) as standards. Culturing medium was Middlebrook 7H9 broth (Sigma-Aldrich) with the addition of glycerol (Sigma-Aldrich) and OADC supplement (Himedia, Mumbai, India). Tested compounds were dissolved in dimethylsulfoxide (DMSO) and diluted with medium to final concentrations 100, 50, 25, 12.5, 6.25, 3.125 and 1.5625 μg/mL. The method used for this assay was microdilution broth panel method. The final concentration of DMSO did not exceed 1% (v/v) and did not affect the growth of mycobacteria. The cultures were grown in Middlebrook 7H9 medium at 37 °C in humid dark atmosphere. The antimycobacterial activity was determined using Alamar Blue colouring after 14 days of incubation as MIC (μg/mL). This evaluation was done in cooperation with Department of Clinical Microbiology, University Hospital in Hradec Kralove (Hradec Kralove, Czech Republic).

#### 3.5.2. Antifungal and Antibacterial *in Vitro* Screenings

Antibacterial evaluation was made using the microdilution broth method in plates M27A-M1 (200 + 10) against 8 bacterial stems from Czech Collection of Microorganisms (Brno, Czech Republic) or clinical isolates from Department of Clinical Microbiology, University Hospital in Hradec Kralove (*Staphylococcus aureus* CCM 4516/08, *Staphylococcus aureus* H 5996/08 methicillin resistant, *Staphylococcus epidermidis* H 6966/08, *Enterococcus* sp. J 14365/08, *Escherichia coli* CCM 4517, *Klebsiella pneumoniae* D 11750/08, *Klebsiella pneumoniae* J 14368/05 ESBL positive, *Pseudomonas aeruginosa* CCM 1961). Mueller Hinton broth was used for the cultivation that was done in humid atmosphere and by 35 °C. The readings were made after 24 and 48 h and MIC was set as 80% inhibition of control. The standards were neomycin, bacitracin, penicillin G, ciprofloxacin and phenoxymethylpenicillin [43].

Antifungal evaluation was also accomplished with microdilution broth method. On the contrary, there was used RPMI 1640 broth with glutamine as medium and conditions were humid and dark atmosphere, pH 7.0 (adjusted with 0.165 M morpholinepropanesulfonic acid, MOPS) and 35 °C. 8 fungal strains were used (*Candida albicans* ATCC 44859, *Candida tropicalis* 156, *Candida krusei* E28, *Candida glabrata* 20/I, *Trichosporon asahii* 1188, *Aspergillus fumigatus* 231, *Lichtheimia corymbifera* 272, *Trichophyton mentagrophytes* 445) together with four antimycotic standards amphotericin B, voriconazole, nystatin and fluconazole. The MIC was set as 80% inhibition of control and readings were made after 24 and 48 h (50% IC, 72 and 120 h for filament fungi) [44].

#### 3.5.3. Study of Photosynthetic Electron Transport Inhibition

The inhibition of photosynthetic electron transport (PET) in spinach chloroplasts was determined spectrophotometrically (Genesys 6, Thermo Scientific, Madison, WI, USA) using an artificial electron acceptor 2,6-dichlorophenol-indophenol (DCPIP) [45]. The rate of photosynthetic electron transport was monitored as a photoreduction of DCPIP. Chloroplasts were prepared from *Spinacia oleracea* L. [46]. The measurements were carried out in phosphate buffer (0.02 mol/L, pH 7.2) containing sucrose (0.4 mol/L), MgCl_2_ (0.005 mol/L) and NaCl (0.015 mol/L). The chlorophyll concentration was 30 mg/L and the samples were irradiated (~100 W/m^2^ with 10 cm distance) with a halogen lamp (250 W). The 4 cm wide water filter was used to prevent warming of the suspension so the temperature did not exceed 22 °C. The studied compound were dissolved in DMSO due to their limited water solubility. The applied DMSO concentration (up to 4%) did not affect the photochemical activity in spinach chloroplasts. The efficiency of studied compounds was expressed as 50% inhibition concentration relative to the untreated control. The standard for these measurements was DCMU (Diurone^®^).

The emission fluorescence spectra of aromatic amino acids were recorded on the fluorescence spectrophotometer F-2000 (Hitachi, Tokyo, Japan). The samples of chloroplast suspensions with and without studied inhibitor were excited at wavelength of 275 nm, excitation slit 20 nm and emission slit 10 nm. The samples were kept in the dark for 2 min prior to the measurement. The phosphate buffer used for dilution of the chloroplast suspension was the same as described above. The compounds were added to chloroplast suspension in DMSO solution due to low aqueous solubility. The DMSO concentration in all samples was the same as in the control (10%). The chlorophyll concentration in chloroplast suspension was 10 mg/L.

## 4. Conclusions

Eighteen *N*-substituted 3-aminopyrazine-2-carboxamides were synthesized in this work with the application of focused field microwave technology. The reaction conditions, which were set experimentally, lead to higher yields and shorter reaction times compared to the conventional heating method. The difference between these conditions is obvious. The temperature reached during the reactions is much higher than the boiling point of methanol and toluene under normal conditions. Also the time is at least two times shorter. All the compounds were purified and then their structure was confirmed by NMR and IR spectra and they were then tested for their potential biological activities.

The experimentally measured lipophilicity was compared to the CS ChemBioDraw Ultra 13.0 program predicted values. The dependence between log*k* and log*P* was linear with one exception where the effect of ionization is appreciable.

The majority of compounds did not show antimycobacterial activity against *M. tuberculosis*. There were only four compounds (**6**‒**9**) with very low activity that were characterized by long or ramified side aliphatic side chain which is probably better for penetration into mycobacterial cell. But the values were significantly lower than the values of standards so it is not possible to predict any structure-activity relationships.

Quite interesting results were found in antifungal assays. Three compounds (**11**‒**13**) showed activities against whole tested fungal spectrum and the activity of compounds **12** and **13** was also better than that of a fluconazole standard. Although there were not enough active compounds in the antibacterial and antifungal screenings, we can propose a hypothesis that the side alicyclic chain is necessary for antifungal activity and that the efficacy rises with increasing number of carbons in the alicyclic ring, *i.e.*, increasing lipophilicity.

PET inhibition screening showed some interesting consequences. There were nine compounds active and activity of the most active substance (compound **9**) expressed by IC_50_ value was 14.3 μmol/L. A quasi-parabolic course was observed for the dependence of PET inhibiting activity on the lipophilic properties of substituents and side chain of the most effective compounds had seven or eight carbons. The active compounds were found to be PS2 inhibitors acting on the donor side of photosystem 2.

Based on the obtained results it can be concluded that the biological activity of the compounds is affected by the presence of secondary amine group bounded to pyrazine ring. If this -NH- fragment is missing, the efficacy would also disappear. This phenomenon can be seen in both evaluations.
